# IgM Promotes the Clearance of Small Particles and Apoptotic Microparticles by Macrophages

**DOI:** 10.1371/journal.pone.0017223

**Published:** 2011-03-23

**Authors:** Michael L. Litvack, Martin Post, Nades Palaniyar

**Affiliations:** 1 Lung Innate Immunity Research Laboratory, The Hospital for Sick Children, Toronto, Ontario, Canada; 2 Physiology and Experimental Medicine, The Hospital for Sick Children, Toronto, Ontario, Canada; 3 Institute of Medical Science, Faculty of Medicine, University of Toronto, Toronto, Ontario, Canada; 4 Department of Laboratory Medicine and Pathobiology, Faculty of Medicine, University of Toronto, Toronto, Ontario, Canada; Chinese University of Hong Kong, Hong Kong

## Abstract

**Background:**

Antibodies are often involved in enhancing particle clearance by macrophages. Although the mechanisms of antibody-dependent phagocytosis have been studied for IgG in greater detail, very little is known about IgM-mediated clearance. It has been generally considered that IgM does not support phagocytosis. Recent studies indicate that natural IgM is important to clear microbes and other bioparticles, and that shape is critical to particle uptake by macrophages; however, the relevance of IgM and particle size in their clearance remains unclear. Here we show that IgM has a size-dependent effect on clearance.

**Methodology/Principal Findings:**

We used antibody-opsonized sheep red blood cells, different size beads and apoptotic cells to determine the effect of human and mouse IgM on phagocytosis by mouse alveolar macrophages. Our microscopy (light, epifluorescence, confocal) and flow cytometry data show that IgM greatly enhances the clearance of small particles (about 1–2 micron) by these macrophages. There is an inverse relationship between IgM-mediated clearance by macrophages and the particle size; however, macrophages bind and internalize many different size particles coated with IgG. We also show that IgM avidly binds to small size late apoptotic cells or bodies (2–5 micron) and apoptotic microparticles (<2 µm) released from dying cells. IgM also promotes the binding and uptake of microparticle-coated beads.

**Conclusions/Significance:**

Therefore, while the shape of the particles is important for non-opsonized particle uptake, the particle size matters for antibody-mediated clearance by macrophages. IgM particularly promotes the clearance of small size particles. This finding may have wider implications in IgM-mediated clearing of antigens, microbial pathogens and dying cells by the host.

## Introduction

Particle clearance is an essential function performed by phagocytes such as macrophages [1]. Antibodies and other soluble innate immune proteins are often involved in opsonizing and promoting the clearance of biological material by macrophages [1], [2], [3]. It is very well established that IgG-coated particles are engulfed efficiently by macrophages [4], [5]; yet, little is known about the role of IgM in particle clearance. Recent studies indicate that shape, but not the size, is critical to the uptake of particles by macrophages [6]. However, the importance of size in antibody-opsonized particle clearance has not been clearly established. The aim of this study is to determine the importance of particle size in IgM-opsonized particle clearance.

Recent studies highlight the importance of natural IgM in opsonizing microbes [7], [8], [9], [10], [11], [12] and dying immune cells for clearance by phagocytes [13], [14], [15], [16]. In this context, natural IgM is considered as an innate immune protein that recognizes non-self particles [14], [17]. Many recent studies also indicate that natural IgM can recognize damaged-self such as late apoptotic or necrotic cells in various tissues [14], [18], [19], [20], [21]. Most of these studies focused on understanding the role of complement activation on IgM-mediated late apoptotic cell clearance [13], [18], [20], [21], [22], [23], [24], [25]. Other in vivo studies show that IgM can protect immune complex-mediated damage in specific tissues [26], [27]. Notably, similar to secretory IgA, IgM can reach mucosal surfaces independently of other complement proteins by Th17 regulated transcytosis [9], [28], [29]. The conundrum is that IgG [4], [5], but not IgM, is considered to mediate phagocytosis. This paradigm led large number of studies to focus on determining the role of IgG, but not of IgM, for phagocytosis.

Our previous studies show that IgM enhances the clearance of late apoptotic cells by macrophages in the absence of any complement activating conditions such as lung inflammation, in vivo [30]. We found that IgM interacts with a population of late apoptotic cells (>∼5 µm) in specific locations (punctate pattern) [30]. In this study, we focus on the role of IgM on small size-late apoptotic cells and blebs/microparticles. Apoptotic cells are known to release blebs and small particles (<5 µm) [31]; however, IgM-mediated clearance of these particles by macrophages has not been studied in detail. Understanding the importance of the clearance of small bioparticles and various types of microparticles in many diseases has gained great interest in the recent years [32], [33], [34]. We hypothesized that IgM mediates the clearance of small particles by macrophages. We have tested this hypothesis using sheep red blood cells (sRBC), various size beads and late apoptotic blebs and microparticles. This study establishes that IgM enhances the clearance of small particle uptake by macrophages. We propose that IgM-opsonized small particle clearance is an alternative pathway to bypass the need for complement activating inflammation-exacerbating pathway that causes excessive tissue damage. Hence, our findings could have a broader biological relevance.

## Results

### IgG, but not IgM, efficiently enhances the uptake of red blood cells by macrophages

**Figure 1:**
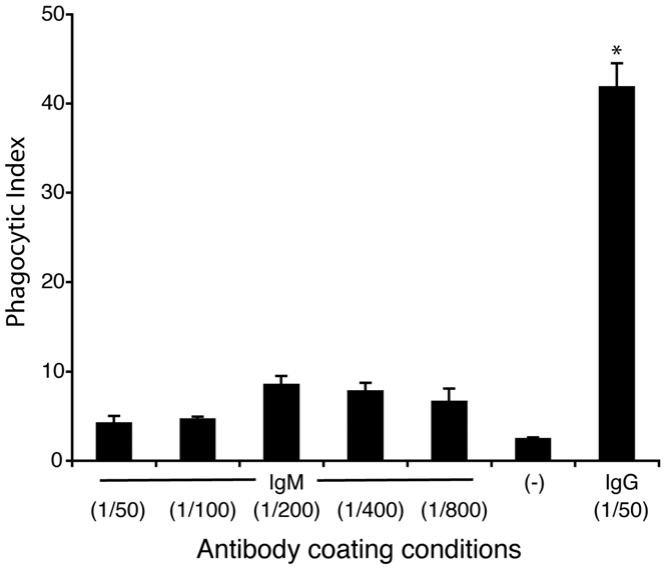
IgG, but not IgM, drastically enhances the uptake of sRBCs by macrophages. Light microscopy analyses show that macrophages effectively phagocytose IgG-coated sRBCs (∼7–10 µm) compared to control sRBC with no coating antibody (p<0.05). IgM-coated sRBCs are not effectively taken up by the macrophages. Although IgM coating conditions shows a small dose-dependence at low concentrations of the antibody, this effect is less likely to represent a biologically significant effect. Phagocytic index represents the number of sRBCs internalized by 100 macrophages. Antibody dilutions used for coating sRBCs are shown within parenthesis. Data are representative of 3 experiments performed on different days with similar results. Error bars are ± SEM from 3 technical replicates.

To evaluate the ability of IgM to regulate the uptake of biological material, we used sRBCs as a model biological particle (∼7–10 µm) for phagocytosis [35]. We coated sRBCs with anti-sRBC IgG or IgM and incubated them with freshly isolated mouse macrophages for 30 minutes. These macrophages readily engulfed IgG-coated sRBCs, displaying a phagocytic index 20-fold greater (42±3) than that of the negative control (2.2±0.2; p<0.05; Fig. 1). IgM-mediated phagocytosis of sRBCs shows a peak at 1/200 antibody dilution (8.4±1); however, considering all the conditions, IgM coating of sRBCs did not show significant phagocytosis of sRBCs by macrophages. Hence, IgG, but not IgM, can greatly facilitate the phagocytosis of these large bioparticles by macrophages.

### IgG, but not IgM, efficiently enhances the binding and uptake of large beads by macrophages

**Figure 2:**
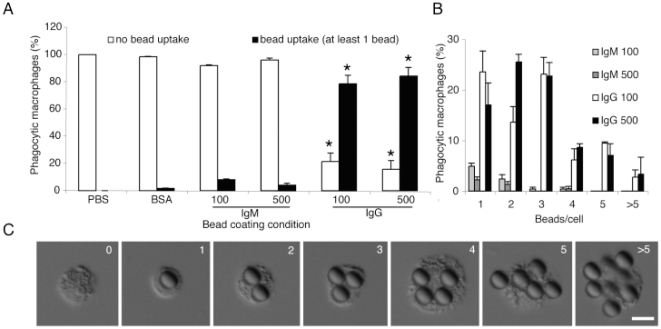
Human IgG, but not IgM, drastically enhances uptake of large beads by macrophages. (A) Macrophages effectively bind and internalize IgG-coated, but not IgM-, BSA-, or PBS-coated, large beads (∼8.31 µm; p<0.05). (B) Counting numbers of “beads/cell” shows that IgG-coated beads are taken up in greater quantities. Only a small number of cells binds or internalize IgM-coated beads. Bead uptake saturates at 100–500 µg/ml IgG-coating concentrations. Percentage values for each IgG and IgM condition will add up to the corresponding values shown in panel A. (C) Differential interference contrast (DIC) images show examples of IgG-coated 8.31 µm large beads that associate with or are engulfed by macrophages. A similar effect was not seen with IgM-, or BSA- or PBS-coated beads. Scale bar is 10 µm.

After identifying that IgM is not effective in directing macrophage engulfment of sRBCs, we tested similar sized beads as a model particle to eliminate the influence of any potential ligands present on the sRBCs that may inhibit IgM-mediated uptake. We compared the uptake by macrophages of large (8.31 µm) beads coated with IgM, IgG, BSA or PBS. Beads were fed to the macrophages for 30 minutes, and attached or internalized beads were assessed by DIC microscopy (Fig. 2A). We found that beads coated with 100 µg/ml or 500 µg/ml of IgG and fed to macrophages were readily bound or phagocytosed by 73.4% or 80.1% of the macrophages, respectively (p<0.05). Bead uptake saturates at 100–500 µg/ml IgG concentrations; hence we used similar concentration range for IgM. However, beads coated with 100 or 500 µg/ml IgM were bound or taken in only by less than 8.7% of macrophages. We further assessed the frequency of macrophages containing multiple beads and categorized the macrophages as a function of ‘beads/cell’ (Fig. 2B). Only the macrophages receiving beads coated with IgG displayed notable frequencies of ‘beads/cell’ exceeding 1 bead/cell (Figs. 2B, C). Both the BSA and PBS control coating conditions did not promote the binding or uptake of beads by macrophages (0 to 1.3%). These data suggest that IgG, but not IgM, has the capacity to efficiently promote macrophage uptake or clearance of large-sized beads or particles.

### IgM preferentially promotes the uptake of smaller particles, whereas IgG enhances the uptake of small and large particles by macrophages

**Figure 3:**
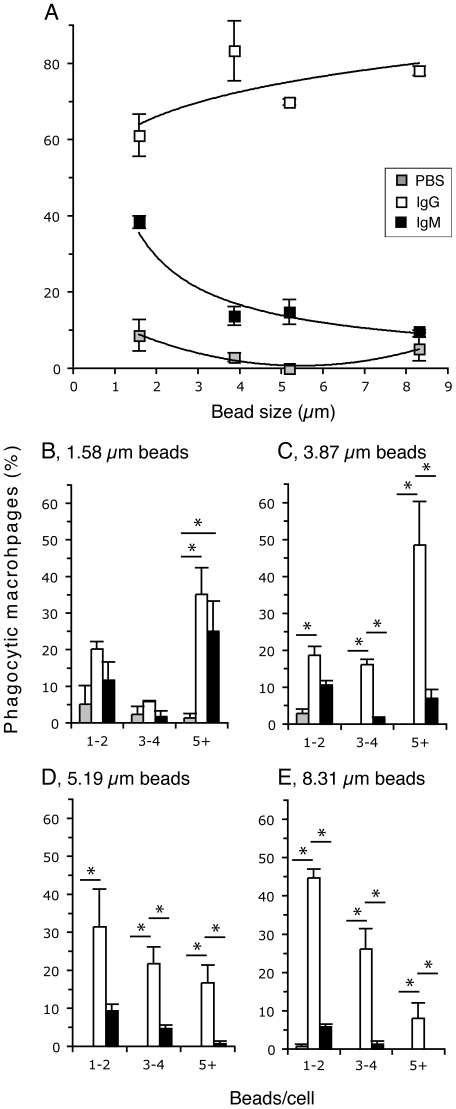
Human IgM preferentially enhances the binding and uptake of smaller-sized particles. Macrophages were incubated with different size beads that are coated with IgM, IgG or PBS. (A) The percentage of macrophages involved in bead binding and uptake compared to bead size and bead coating condition. Phagocytic macrophages are the cells that contained at least one bead. Non-linear regression analyses between phagocytic microphage (%) and bead diameter: IgG (y = 60.1 x^0.1354^; r^2^ = 0.49) and IgM (y = 51.6 x^−0.815^; r^2^ = 0.94). These two regression lines are different from zero and to each other (p<0.05). PBS condition does not fit a similar mathematical equation. (B–E) Phagocytic macrophages shown in (A) were examined and counted for the number of beads bound and internalized under varying protein coating conditions and the bead diameter. The percentages of phagocytic macrophages were plotted separately for beads with (B) 1.58 µm, (C) 3.87 µm, (D) 5.19 µm and (E) 8.31 µm in mean diameter. X-axis is the same for charts B–E: 1–2, one to two; 3–4, three to four; 5+, 5 or more beads/cell. Note: Only the phagocytic macrophages are shown in the bar charts; hence, the values for each condition shown in (B–E) will add up to the corresponding values shown in (A); *, p<0.05 (Tukey's test).

We compared a range of bead sizes to determine if IgM could exert phagocytic functions on smaller particles. We tested four sized beads with mean diameters of 1.58 µm, 3.87 µm, 5.19 µm and 8.31 µm. Macrophages were incubated with the protein-coated beads for 30 minutes. These cells were scored for bead binding and uptake as a function of particle size and protein condition (Fig. 3A). This analysis revealed that IgG effectively directs macrophage binding and uptake of both small and large particles. Small particles (1.58 µm beads) coated with IgG are bound or taken up by 61.1% of macrophages, while this is even greater, at 78.1% when the IgG-coated particles are larger (8.31 µm). IgG shows a positive trend in promoting the clearance of larger particles (Fig. 3A). It is interesting to note that IgM however favours the clearance of the small particles. Small (1.58 µm beads) and large (8.31 µm) particles coated with IgM are bound or taken up by 38.3% and 9.69% of the macrophages, respectively. Non-linear regression analyses show the relationship between these two parameters (IgG: y = 60.1 x^0.1354^; r^2^ = .49; IgM: y = 51.624 x^−0.815^; r^2^ = 0.94); their slopes have non-zero values and these two regression lines are different from each other (p<0.05). Therefore, these data confirm that IgM-mediated binding and uptake is inversely related to particle size.

We further assessed the bead binding and uptake per cell amongst each protein condition for each of the four bead sizes. Macrophages that contained 1.58 µm beads coated with IgM showed a notable frequency of cells involved in multiple bead binding and uptake (Fig. 3B); whereas a similar analysis for larger beads 3.87 µm (Fig. 3C), 5.19 µm (Fig. 1D), and 8.31 µm (Fig. 3E) indicated a very low frequency of macrophages involved in multiple IgM-coated bead binding and uptake. Conversely, beads coated with IgG were bound or phagocytosed in multiples across the bead size range (Fig. 3B–E). Macrophages incubated with PBS-coated beads did not show notable levels of multiple bead binding and internalization. Taken together these data suggest that IgG directs the uptake by macrophages of a range of particle sizes from small to large, but IgM preferentially directs macrophage uptake of small particles only.

### IgM enhances the clearance of small particles in a concentration dependent manner

**Figure 4:**
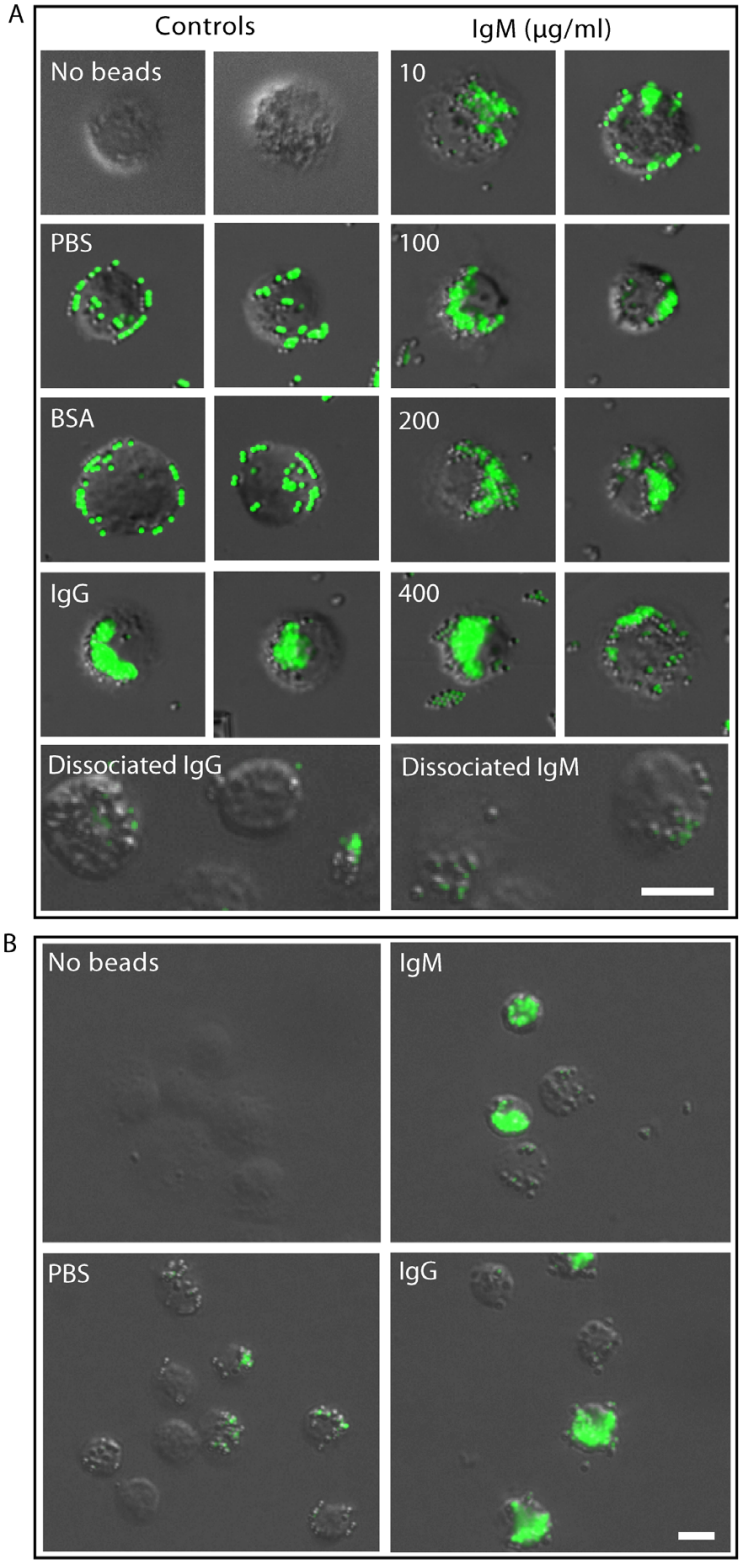
Epifluorescence microscopy reveals that both human and mouse IgM enhance the binding and uptake of small particles by macrophages. (A) Mouse macrophages incubated with human IgM-, human IgG-, BSA-, or DPBS-coated 1 µm beads show distinct fluorescence bead binding or internalization patterns. Left panel shows macrophages and their binding and uptake of beads coated with PBS, BSA (100 µg/ml), IgG (100 µg/ml) or dissociated IgG (100 µg/ml). Right panel shows the uptake of IgM (0–400 µg/ml) or dissociated IgM (100 µg/ml)-coated beads by representative macrophages. Coating of beads with increasing concentrations of IgM shows increasing fluorescence in the macrophages. Dissociating both IgG and IgM eliminates antibody-dependent uptake of these beads by macrophages. Numerical values denote the concentrations of IgM (µg/ml) used for coating the beads. (B) Macrophages phagocytose mouse IgG (100 µg/ml) and mouse IgM (100 µg/ml)-coated small beads (1 µm). Both human (A) and mouse (B) antibodies show similar effects. Scale bar is 10 µm.

Following our finding that IgM preferentially enhances the clearance of small particles by macrophages, we sought to determine whether IgM promotes the clearance of beads in a dose-dependent manner. We used fluorescent beads 1 micron in diameter, coated the beads with varying concentrations of IgM (0, 10, 100, 200 and 400 µg/ml) and incubated the beads with macrophages for 30 minutes. Epifluorescence microscopy analyses showed that compared to negative controls (PBS, BSA) both IgM and IgG (100 µg/ml) influenced the pattern of uptake of the beads by macrophages (Fig. 4A). Cells incubated with IgM- or IgG-coated beads displayed dense irregular fluorescent masses well within the perimeters of the cells, whereas cells incubated with BSA or PBS alone displayed fluorescent beads mostly near the perimeter of the cells. They exhibited mostly single point fluorescence around the cells (Fig. 4A). Microscopy images further show that coating the beads with increasing amounts of IgM increased the degree of fluorescence detected in the macrophages.

To verify that the intact antibodies are necessary for bead uptake, 100 µg/ml IgG and IgM were treated with DTT, boiled and coated onto the beads. These beads were washed and incubated with macrophages; however, macrophages, did not take up these beads to any significant degree (Fig. 4A). These results show that intact IgG and IgM are necessary for promoting the phagocytosis. To further show that mouse antibodies behave similar to human antibodies (Fig. 4A), we repeated the bead uptake experiments using mouse IgG, mouse IgM and mouse macrophages. Both mouse IgG and IgM promoted the uptake of small beads by mouse macrophages (Fig. 4B). Therefore, we conclude that both human and mouse antibodies have similar properties in promoting bead uptake by mouse macrophages.

**Figure 5:**
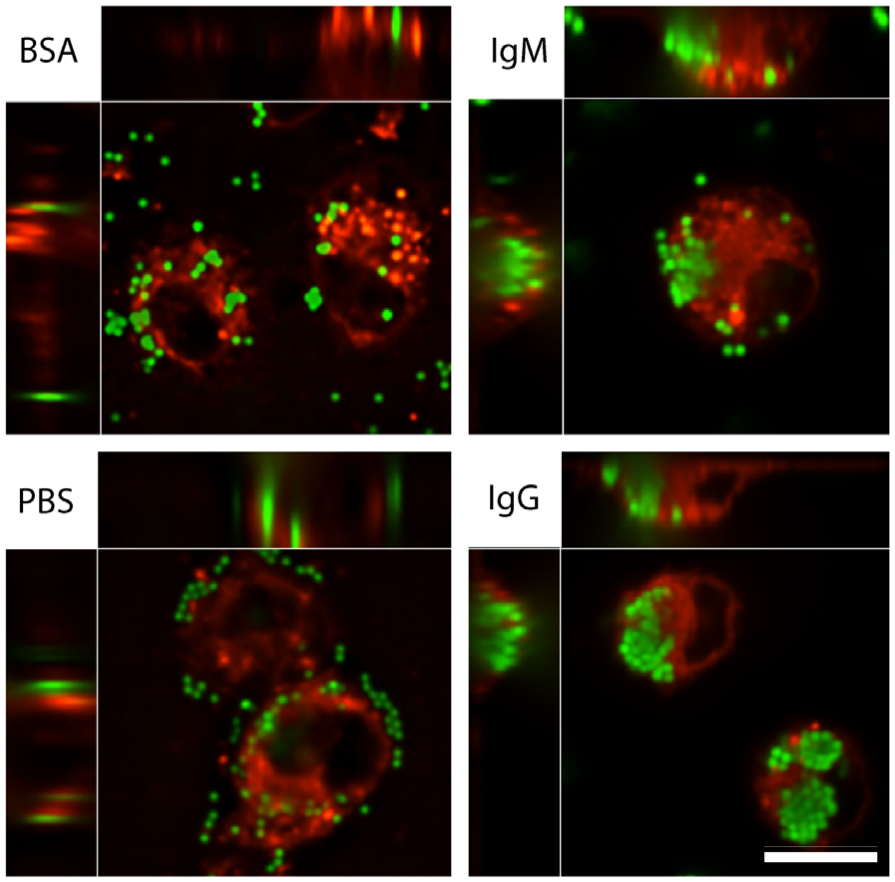
Confocal microscopy images show that macrophages internalize large numbers of human IgG- and IgM-coated beads. Confocal images show that macrophages internalize only a few BSA- or PBS-coated beads (1 µm); these beads are primarily bind to macrophages. In contrast, macrophages effectively internalize large numbers of IgG- and IgM-coated beads. Most of these beads are found as 3-D masses within the macrophages. Top and left strips in each panel show different views (X, Y, Z) of the same cells at a confocal plane. Scale bar is 10 µm.

Although epifluorescence microscopy is routinely used for bead uptake experiments whether beads are internalized by the macrophages cannot be precisely established by this procedure. Therefore, we used confocal microscopy to determine the nature of bead uptake by macrophages. Confocal analyses showed the presence of large numbers of densely populated beads within the macrophages when IgG- or IgM-coated beads were used, whereas control BSA- and PBS-coated beads remained sparsely populated within the cells (Fig. 5). Overall, these data suggest that the protein coating of the beads by IgM and IgG directs internalization of these small particles by macrophages.

**Figure 6:**
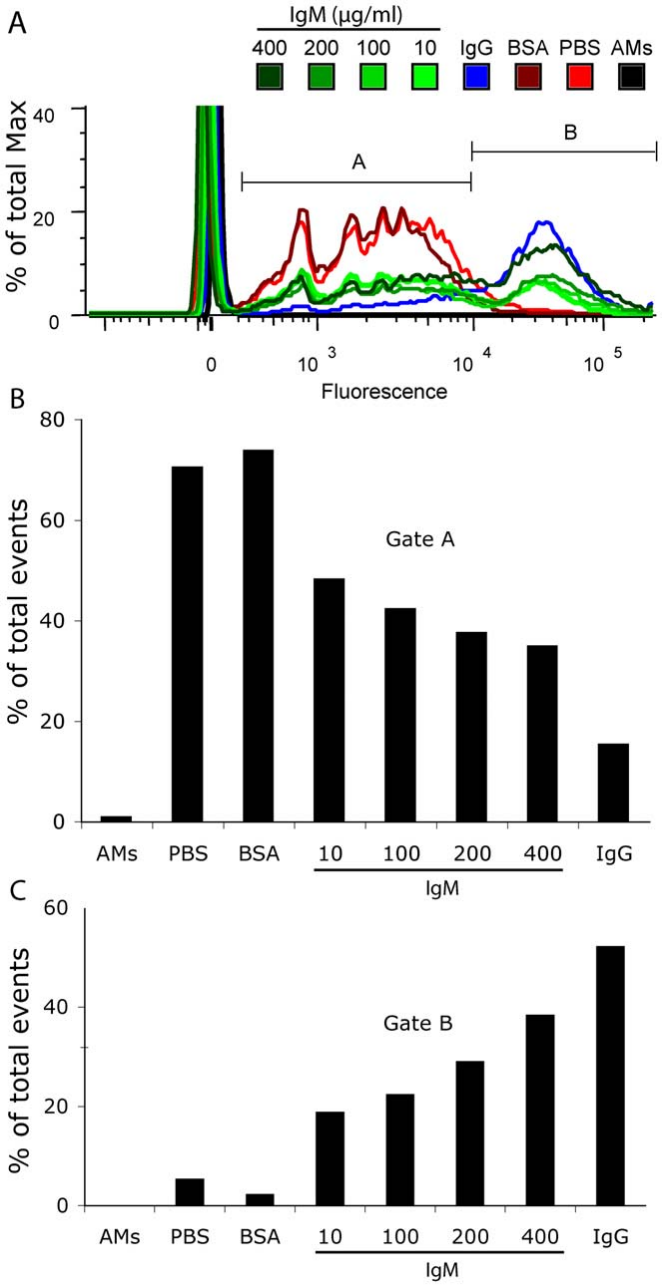
Flow cytometry reveals that IgM enhances the binding and uptake of small particles by macrophages in a concentration-dependent manner. (A) Overlay comparison (% of Max) of IgM-coated (10, 100, 200, 400 µg/ml), IgG-coated (100 µg/ml), BSA-coated (100 µg/ml) or DPBS-suspended beads. IgM conditions are represented with varying hues of green lines, IgG in a blue line, BSA in dark red, PBS in bright red, and macrophages alone with a black line. Gates A and B partition cells containing beads to resolve the population of cells containing few beads from those with many beads. (B) Gate A represents the population of cells (% of total events) with few beads as indicated by lower fluorescent values. (C) Gate B represents the population of cells (% of total events) with many beads as indicated by higher fluorescent values.

Counting all the beads present in clusters within the cells is not precise when smaller beads (e.g., 1 µm) are used; hence, to quantify the IgM-mediated effort on small bead clearance we repeated representative fluorescence microscopy experiments and analyzed the macrophages using flow cytometry (Fig. 6). We found that coating beads with IgM caused a concentration-dependent increase in bead binding and uptake by macrophages compared to negative control beads coated with either BSA (100 µg/ml) or PBS only (Fig. 6A). Macrophages bound and phagocytosed IgG-coated beads, as was expected from our fluorescence microscopy experiments outlined above; however, to more clearly understand the effect of IgM on the bead uptake, we segregated the flow cytometry data into distinct gates. Gate A indicates that both PBS and BSA control conditions had most of the cells within the lower fluorescence gate, indicating the presence of a few beads per cell under these conditions (Fig. 6B). Conversely, gate B (Fig. 6C) best illustrates the concentration dependent effect of IgM on enhancing the binding and uptake of small particles by macrophages. As the IgM concentration increases the percent of total events counted within that gate also increases proportionally, indicating that IgM promotes binding or internalization of relatively larger number of beads by most of the macrophages. Taken together, these data further shows that IgM increases the ability for macrophages to phagocytose micron-sized particles in a concentration-dependent manner.

### IgM binds to small-size late apoptotic cells and vesicle-like small apoptotic particles

**Figure 7:**
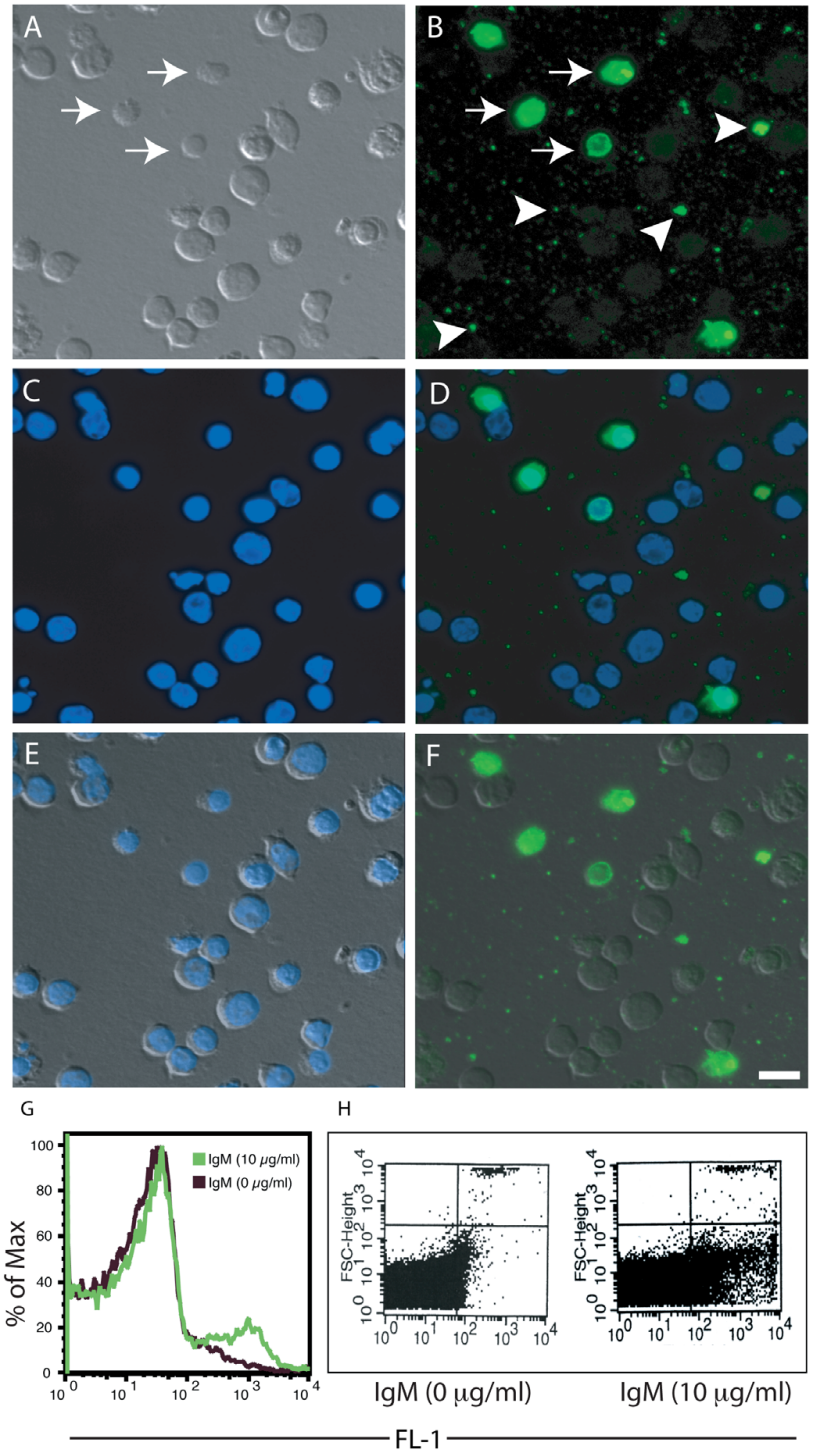
Human IgM preferentially binds to a subpopulation of late apoptotic cells and vesicle-like small apoptotic particles (v-SAPs) or apoptotic “microparticles”. (A) Differential interference contrast (DIC) microscopy image. (B) Epifluorescence microscopy image showing the preferential interaction between IgM (green) and small apoptotic cells (arrow) and microparticles (arrowheads). (C) Fluorescence image showing that these cells still contain DNA stained blue with DAPI. (D) Overlay of (B) and (C) shows the locations of IgM binding in relation to the location of the nuclei. (E) Overlay of (A) and (C) shows the locations of nuclei in these cells. (F) Overlay of (A) and (B) shows IgM also binds to larger cells as small faint punctate dots. Overall, these images show that IgM avidly binds to a subpopulation of relatively smaller cells with smooth regular surfaces (arrows) and v-SAPs or microparticles (arrowheads). Scale bar is 10 µm. (G–H) Flow cytometry showing that IgM binds small size late apoptotic cells and microparticles or v-SAPs. (G) Flow cytometry shows that large amounts of IgM can bind to smaller apoptotic cells using a voltage setting that is sensitive to events measured at 2 µm or greater. (H) Flow cytometry confirming that IgM can bind to v-SAPs using a voltage setting that is sensitive to events measured at 0.5 µm or greater.

Natural IgM preferentially binds late apoptotic cells [20], [22]. Our recent studies show that purified IgM and IgM present in the normal human serum bind to late apoptotic cells, particularly to specific regions of the cells [30]. These data illustrated a general increase in the number of cells to which IgM binds as cells progressed through to the later stages of apoptosis. During apoptosis, dying cells can release microparticles similar in size to the small beads (Figs. 1, 2, 3, 4, 5, 6). Thus, to evaluate a potential biological relevance for the data presented above, we examined late apoptotic cells incubated with IgM and probed the cells with a fluorescent antibody against IgM using fluorescence microscopy. We found that IgM preferentially binds well to a subpopulation of the late apoptotic cells that appear smaller and smoother in relative size and texture (Figs. 7A–F). The characteristic features suggest that they are late apoptotic bodies. Additionally, we observed IgM bound to numerous microparticles (<2 µm) or vesicle-like very small apoptotic particles (v-SAPs) that were released locally by apoptotic cells (Figs. 7A–F).

To verify that IgM was binding to these smaller apoptotic cells and particles, we also performed flow cytometry on these late apoptotic cells with a low event size cut-off (2 µm or greater; Fig. 7G). These results indicated that IgM specifically binds to small size cells. This flow cytometry data would correspond to the highly labeled small size cells seen by fluorescence microscopy (Figs. 7A–F). To verify the interaction between IgM and v-SAPs by flow cytometry, we further reduced the event size cut-off to 0.5 µm or greater. The data showed that IgM indeed bound to v-SAPs present in late apoptotic cell preparations (Fig. 7H). In a representative experiment, gated events from control preparations with no IgM displayed 0.32% of total events while in preparations containing 10 µg/ml IgM 8.17% of total events were counted within the same gate (>25-fold difference). Therefore, taken together our results indicate that IgM binds to small-sized late apoptotic cells and v-SAPs.

### IgM increases the uptake of v-SAP-coated beads by macrophages

**Figure 8:**
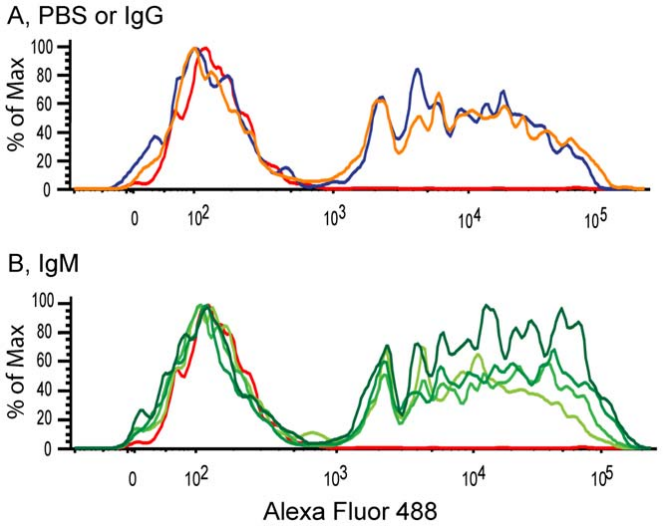
Flow cytometry shows that human IgM increases the uptake of fluorescent beads coated with v-SAPs by alveolar macrophages. Fluorescent beads coated with v-SAPs were incubated with (A) PBS or IgG (100 µg/ml) or (B) with increasing concentrations of IgM (10, 100, 200, 400 µg/ml). Macrophage negative control is represented by the red line in both (A) and (B). In (A), the blue line represents PBS and the orange line is IgG. In (B), increasing concentrations of IgM are represented by the increasing darkness of the green lines.

The v-SAPs are small biological particles that can quickly be processed by macrophages. Thus to ascertain v-SAP uptake, we incubated v-SAPs with the fluorescent beads, allowed IgM or IgG to bind to these particles and fed them to macrophages. This method permitted us to measure and compare the effect of IgM on the internalization of the v-SAP-coated beads. We used flow cytometry to compare PBS and IgG control conditions (Fig. 8A) with various concentrations of IgM (10–400 µg/ml; Fig. 8B). Flow cytometry graphs show that IgM increases in the uptake of v-SAP-coated beads in a concentration-dependent manner; moreover, IgG did not generate a notable increase in clearance compared to the PBS control (Fig. 8A). Thus, our data suggests that IgM specifically bound vSAP-coated beads and facilitates their clearance by macrophages.

## Discussion

The binding and uptake of particles by macrophages are essentials functions attributed to these phagocytic cells of the innate immune system. The clearance of various bioparticles is a primary responsibility of macrophages [36], [37], [38], [39], [40]. Understanding this process is not complete without describing the parameters that influence particle uptake. In a recent study, the geometry of target particles has been shown to be critical to the phagocytic function of macrophages [6]. Here we show that particle size plays a central role regulating antibody-dependent clearance of particles (Figs. 1, 2, 3, 4, 5, 6). We compared the uptake of different sized particles opsonized with IgG or IgM by macrophages. IgM preferentially enhances the uptake of small particles (∼1 µm). We further identify a biological relevance to our findings by illustrating that IgM binds to small apoptotic cells and very small microparticles released from these apoptotic cells (Fig. 7). Furthermore, IgM promotes the clearance of v-SAP-coated beads by macrophages (Fig. 8). Our study therefore describes two novel functions for IgM – (i) antibody-mediated small particle clearance and (ii) binding to small apoptotic cells and microparticles, and the clearance of microparticle beads by macrophages.

Macrophages phagocytose antibody-coated particles that are more than 1 µm in diameter [35], [36]; and to identify the role of IgM in particle clearance, we used antibody opsonized sheep red blood cells as a model biological target particle for phagocytosis (Fig. 1). This is a well-established model of particle uptake that is commonly used to examine phagocytic events [35], [41]. Our data illustrates that IgG-opsonized sRBCs, but not varying dilutions of anti-sRBC IgM, are readily engulfed by macrophages within 30 minutes of feeding. Thus, the function of IgM in antibody-mediated engulfment is likely distinct from that of IgG, and may have a different biological relevance. Similar experiments using uniformly sized beads that are similar in size range as the sRBCs (Fig. 2), again show that IgG, but not IgM, facilitates the engulfment of these large beads (∼8.31 µm) at both low and high concentrations. Both of these experiments indicate that IgM does not mediate clearance in the same manner as does IgG.

We then address the effect of particles size on uptake by using various sized beads ranging from 1.58 µm to 8.31 µm opsonized with IgG or IgM (Fig. 3). The data illustrates that IgM opsonization preferentially mediates the clearance of the smaller particles, whereas IgG opsonization mediates the clearance of particles of various sizes, showing a positive trend for enhancing the clearance of larger particles. A previous study suggests that IgG preferentially enhances the uptake of mid-size (2–3 µm) particles, but not larger particles [42]. However, the ability of IgG to enhance the phagocytosis of large beads and sRBCs has been well established [4], [5], [35]. Our data (Figs. 1, 2, 3, 4, 5, 6) are consistent with the fact that IgG internalizes small and large particles.

After identifying various particle sizes and establishing that IgM opsonization enhances the clearance of the small particles, we tested a variety of IgM concentrations to more clearly understand the effects of IgM opsonization on small particle clearance. We used fluorescent microscopy (Figs. 4, 5) and flow cytometry (Fig. 6) to evaluate the concentration effect of IgM opsonization on small particle (1 µm) uptake by macrophages [40], [43]. Fluorescent microscopy enabled us to qualitatively compare the pattern of small particle uptake caused by IgM opsonization where as confocal images confirmed that IgM promote the phagocytosis of these beads. This analysis revealed a concentration dependent effect, where increased numbers of particles are internalized by macrophages when the particles are coated with higher concentrations of IgM. It is known that human, rabbit and mouse antibodies can support the uptake of particles by mouse macrophages [35]; however, we tested species matched antibodies to confirm the effect of IgM on phagocytosis. Like human IgM, mouse IgM also enhanced the phagocytosis of small beads, but not large particles by mouse macrophages (Figs. 1, 4).

After establishing that IgM opsonization enhances small particle clearance, we considered a biological situation. Recently, we have identified that IgM can enhance the clearance of apoptotic cells in the lungs of mice by binding directly to late apoptotic cells [30]. Moreover, IgM has been implicated indirectly in apoptotic cell clearance – a direct effect was not shown [13], [21], [22], [44], [45]. This illustrates the importance for understanding the role IgM plays in particle clearance. Furthermore, the role played by natural IgM in a variety of diseases including tissue injury, autoimmunity, cancer and immune surveillance, has gained considerable interest recently [17], [19], [20], [46]. Interestingly, IgM binds to small late apoptotic cells and to vesicle-like small apoptotic particles (Fig. 7), and enhances the clearance of beads coated with these small apoptotic particles (Fig. 8). Thus, the IgM-mediated size-restrictive clearance is likely to functions as a means of achieving apoptotic cell clearance without promoting inflammation.

In addition to our proposed biological function of size-restricted IgM-mediated particle clearance, some studies suggest that IgM is involved in antibody-mediated engulfment of microbes [8], [9], [12]. Furthermore, use of nanoparticles and microparticle for immune therapies and vaccines is becoming an important aspect of treating several diseases [34]; however, the immune molecules that regulate the clearance and delivery of these antigens have not been clearly established. The accumulation of immune complexes, dying cells, apoptotic blebs and microparticles in tissues and organs are root causes for many autoimmune diseases [32], [33], [47], [48], [49]. Therefore, in addition to determining the roles of other IgM-like innate immune proteins such as lung surfactant protein D [1], [2], [43], [50], [51], the complete understanding of IgM-mediated apoptotic particle clearance is essential to effectively treat and control diseases of biological particle accumulation, autoimmunity (e.g., SLE or rheumatoid arthritis), infection and inflammation.

## Materials and Methods

### Ethics statement

All animal work was completed with the approval of the Research Ethics Board of the Hospital for Sick Children, Toronto, Ontario, Canada (No: 1000008179).

### Animals

Six to twelve week old CD1 mice were purchased from Charles River Canada (Saint-Constant, Québec) and maintained at the Laboratory Animal Services (LAS) at the Hospital for Sick Children, Toronto.

### Reagents and buffers

All reagents or antibodies were purchased from Sigma-Aldrich Canada Ltd. (Oakville, Ontario) unless otherwise stated. Key antibody reagents used in this study are: Soluble IgM purified from pooled human plasma. Sigma-Aldrich used selective protein precipitation and gel filtration techniques for IgM purification. Identity and purity of the IgM was established by immunoelectrophoresis (IEP) and high-pressure liquid chromatography (HPLC). Electrophoresis of the immunoglobulin preparation followed by diffusion versus anti-human whole serum resulted in a single arc of precipitation (product #I8260); FITC-labeled goat-anti-human IgM (product #F6255); mouse anti-sRBC IgM (#CL9000-1, Cedarlane); rabbit anti-sRBC IgG (#ICN55806; ICN/Cappel West Chester, PA, USA); human IgG (#I8640). Hanks buffered saline solution (HBSS, Gibco) was supplemented with 3 mM CaCl_2_ unless otherwise indicated. Cell media used include RPMI (Wisent) and macrophage serum-free media (M-SFM) from Gibco (product #12065). Both media were supplemented with 10 U/ml penicillin, 10 µg/ml streptomycin and 1 µg/ml of Fungizone (Amphotericin B - all from Gibco).

### Generation of apoptotic cells and IgM binding

The human Jurkat T-cells were purchased from ATCC (clone E6-1 ATCC# TIB-152) and cultured in RMPI media (Wisent) supplemented with heat inactivated 10% (v/v) FBS (Gibco), 10 U/ml penicillin and 10 µg/ml streptomycin (Gibco; in complete culture media) at 37°C with 5% (v/v) CO_2_. For apoptosis assays, Jurkat cells were adjusted to a concentration of 1 × 10^6^ cells/ml in 2 ml of HBSS. Cells were seeded in 12-well plates on sterile coverslips. Apoptosis of the cells was induced by 30 seconds of UV-irradiation (254 nm) with a Stratalinker 2400 crosslinker (Stratagene) using the default power settings (120,000 µJ/cm^2^). The buffer was removed and replaced with HBSS buffer (control) or HBSS with IgM or FITC-labeled human IgM (10 µg/ml) and the cells were incubated for 24 h at 37°C with 5% CO_2_.

### Fluorescence microscopy

Following the 24 hour incubation (with no IgM controls or 10 µg/ml human IgM), cells were briefly washed and fixed with 2% (v/v) PFA. Cells were then probed with FITC conjugated goat anti-human IgM antibody (10–20 µg/ml) in the plate wells and washed. Finally, mounting media containing 4′, 6-diamidino-2-phenylindole (DAPI) was added, incubated and slides were adhered to a glass microscope slide, dried and examined with an epifluorescence microscope (Leica, Bannockburn, IL. U.S.A). Images were created using the OpenLab (Improvision, U.K.). The control slides (no IgM, but with FITC conjugated goat anti-human IgM antibody) were used for setting the background, in which no fluorescence signal was detectable. Using the same setting, experimental slides were imaged.

### Flow cytometry

After the induction of apoptotic cells, cells were incubated with no IgM buffer control or 10 µg/ml human IgM for 24 h in Eppendorf tubes, washed, suspended in HBBS and probed with a FITC conjugated goat anti-human IgM antibody (10–20 µg/ml; pre-centrifuged at 10,000 × g; 0.22 µm filter). Cells in suspension were fixed in 1–2% (v/v) PFA. Fluorescence intensities of cells were acquired with a FACScan flow cytometer (Becton Dickinson, Mississauga ON). Voltages were adjusted for sensitivity to particles greater than or equal to 2 µm or 0.5 µm. Data was analyzed with Flowjo (Tree Star Inc., Ashland OR. U.S.A.). See bead uptake section for additional flow cytometry information.

### Alveolar macrophage cell culture

Alveolar macrophages were obtained by bronchoalveolar lavage (BAL) of mice as previously described [40]. Briefly, mice were sacrificed and their abdominal cavities were opened, and exsanguinated, revealing the lungs. The lungs were cyclically inflated and deflated 3 times with 1 ml of DPBS (Gibco) using a 1 ml syringe and a BD Angiocath (BD Bioscience) inserted into the exposed trachea. This was repeated 5 times for a total lavage fluid volume of 5 ml. Lavage fluid from 2 or more mice was pooled and kept on ice until centrifugation for 20 min at 400×g at 4°C. Cell pellets were treated with sterile water for 10 seconds to lyse residual erythrocytes followed by a 1∶9 dilution of the water with M-SFM cell culture media. The cells were seeded equally into chambers of 8-well chamber slides (BD Falcon, REF# 354118) and macrophages were allowed to adhere to the glass slides for a minimum of 30 minutes at 37°C. Cells were then washed with sterile DPBS and incubated further in M-SFM for up to 4 h then used for uptake experiments. For confocal microscopy experiments, macrophages were labeled with PKH 26 dye (Sigma).

### sRBC phagocytosis

To coat the sheep red blood cells with antibody, 200 µl of cells per condition were centrifuged for 10 s at 2000×g. The cells were washed in 1 ml of DPBS and pelleted again with a 10 s centrifugation. The washed pellets were resuspended in DPBS containing mouse anti-sheep erythrocyte IgM at the necessary dilution (1/50, 1/100, 1/200, 1/400, or 1/800), rabbit anti-sheep erythrocyte IgG (1/50) or buffer alone. The sRBCs were incubated in a Thermomixer R (Eppendorf) for 2 h at 37°C with continuous shaking at 900 RPM. Following incubation, sRBCs were washed 2–3 times with DPBS and resuspended in M-SFM. From this, 100 µl of sRBCs was introduced to each wells of an 8-well chamber slide already containing adherent macrophages obtained and cultured as described above. One well remained free of sRBCs to serve as double negative control. The cells were co-incubated for 30 min at 37°C with 5% (v/v) CO_2_. Following this incubation slides were placed on ice for 10 minutes to stop phagocytosis. Chambers were carefully washed by the addition and removal of 200 µl of DPBS. Residual sRBCs were lysed with the addition of 200 µl of sterile water for 30 s. Following sRBCs lysis, solution in each well was replaced with 200 µl of DPBS to maintain the vitality of the cells while immediately viewing and photographing the live cells with a light microscope and a digital camera. The sRBC phagocytic index represents the number of sRBC internalized per 100 macrophages. The values are expressed as the mean ratio ± SEM for an n = 3.

### Bead uptake

Yellow-green fluorescent or colourless beads (Polysciences, Inc., Warrington PA, USA; and Bangs Laboratories, Fishers, IN, USA), ranging in sizes from 1 to 8.31 µm in mean diameter, were suspended in DPBS then briefly sonicated to generate a uniform suspension of single beads as previously described [40], [43]. Beads for each size and condition were centrifuged to pellet with a tabletop microcentrifuge. Beads were incubated in 50 µl of human IgM or IgG (10, 100, 200, 400 µg/ml), BSA (100 µg/ml), or DPBS for 75–90 minutes at 37°C on a Thermomixer R (Eppendorf) shaking at 900 RPM. In some experiments, 100 µg/ml IgG and IgM were incubated at 37°C for 30 min in the presence of 50 mM dithiothreitol (DTT) then boiled for 30 min in the same buffer and cooled back to 37°C before using them for coating the beads. Following this incubation, beads were centrifuged as previously described and washed 1–2 times with 50 µl of DPBS. Protein-coated and control beads were resuspended into 100 µl of M-SFM. The beads were added to macrophages obtained and seeded into the wells of 8-well chamber slides as described above. The slides were centrifuged on a swinging-arm plate rotor at 100×g for 2 min to ensure beads made contact with macrophages in a time-synchronized manner. The slides with beads and cells were incubated for 30 min at 37°C with 5% CO_2_ (v/v) in M-SFM. Following incubation, media was removed and cells were washed extensively 2–3 times with sterile DPBS. We have also tested the assay using RPMI supplemented with heat inactivated 10% (v/v) FCS instead of M-SFM. Both of these media showed similar IgM-mediated binding and uptake of small particles (data not shown). However, we used M-SFM to eliminate any potential confounding effects exerted by the antibodies present in the heat inactivated FCS.

For flow cytometry experiments, cells were treated with Accutase and/or Trypsin (Sigma) to reduce adherence for 10–20 min at 37°C+5% CO_2_ (v/v). Cells were then scraped off the glass slide surface with a micropipette tip. Cells were immediately added to 4% (v/v) PFA for fixation and incubated overnight at 4°C in the dark. Cells were pelleted and resuspended in DPBS and analyzed with a BD FACS ARIA-II. Cells examined by fluorescence or light microscopy (Leica, Bannockburn, IL. U.S.A) were fixed with 4% (v/v) PFA immediately after through DPBS washes and overnight storage at 4°C in the dark. Slides were mounted with Dako fluorescent mounting media and a glass coverslip and allowed to dry overnight. Images were processed using the OpenLab or Volocity (both from Improvision, U.K.). For each condition, macrophages were counted for 2–5 replicate experiments (100–500 cells/condition, and at least 50 cells for negative controls). These slides were also examined with a spinning disc confocal microscope (Carl Zeiss Canada, Toronto) and analyzed in three dimensions using Z-stack imaging Volocity (Improvision, U.K.).

For some experiments, 24 h apoptotic cell suspensions were centrifuged for 10 min at 400×g. The supernatant was removed and recentrifuged at a high speed (25,000×g for 20 min) to pellet microparticles. These microparticles were allowed to coat 1 µm green fluorescent beads in a shaker for 2 h, washed (10 min at 400×g) and further incubated with different concentrations of IgM (10, 100, 200, 400 µg/ml), IgG (100 µg/ml) or Tris buffer for 60–90 minutes at 37°C, followed by a 20–30 incubation on ice. Unbound proteins were removed by washes (10 min at 400 × g), and the beads were incubated with alveolar macrophages in M-SFM for 30 min at 37°C. The cells were processed for flow cytometry as described above.

### Statistical analysis

Where statistical analysis was deemed appropriate to evaluate differences amongst conditions or their respective controls, an ANOVA was used for multiple means comparison with a Tukey's HSD correction or a Dunnett's control comparison test (where necessary) using the JMP statistical package (SAS, North Carolina). Statistically significant data are noted by asterisks (*, p<0.05). To compare line functions, regression analysis was performed using the JMP or the Prism statistical package (GraphPad, San Diego, California). All data presented in this paper is representative of 2–5 replicate experiments and figures were organized using Microsoft Excel and Adobe Illustrator.
